# Knowledge and perception on antimicrobial resistance and antibiotics prescribing attitude among physicians and nurses in Lambaréné region, Gabon: a call for setting-up an antimicrobial stewardship program

**DOI:** 10.1186/s13756-022-01079-x

**Published:** 2022-03-03

**Authors:** Bayode Romeo Adegbite, Jean Ronald Edoa, Frieder Schaumburg, Abraham S. Alabi, Ayola Akim Adegnika, Martin P. Grobusch

**Affiliations:** 1 Centre de Recherches Médicales en Lambaréné and African Partner Institution, German Centre for Infection Research (CERMEL), Lambaréné, Gabon; 2 Centre of Tropical Medicine and Travel Medicine, Department of Infectious Diseases, Amsterdam University Medical Centers, location AMC, Amsterdam Infection and Immunity, Amsterdam Public Health, University of Amsterdam, Meibergdreef 9, 1105 AZ Amsterdam, The Netherlands; 3 Institute of Medical Microbiology, University Hospital Muenster, Muenster, Germany; 4 Institut Für Tropenmedizin, Universität Tübingen and German Centre for Infection Research, Tübingen, Germany; 5 Department of Parasitology, Leiden University Medical Centre, Leiden, The Netherlands; 6 Masanga Medical Research Unit, Masanga, Sierra Leone; 7 Institute of Infectious Diseases and Molecular Medicine, University of Cape Town, Cape Town, South Africa

**Keywords:** Antimicrobial resistance, Healthcare workers, Survey, Gabon, Antimicrobial stewardship

## Abstract

**Background:**

Africa is challenged by the emergence of antimicrobial resistance (AMR). In order to improve patient management and to optimise approaches to curb the spread of antimicrobial resistance, we examined knowledge and perceptions of AMR and antibiotics prescription practices of HCW (healthcare workers) in Lambaréné, Gabon.

**Methods:**

We conducted a self-administered, questionnaire-based survey in HCW at the regional referral hospital, a medical research centre, and peripheral health care facilities. The proportions of correct responses to questions were determined and compared between physicians and nurses using Fisher’s Exact test.

**Results:**

A total of 47 HCW took part in the survey. Of those, 64% (30/47) recognised antibiotic resistance as a major public health issue in Gabon, but only 14/47 (30%) recognised it as a problem in their health facility. Of note, 37/47 (79%) recognised excessive use of antibiotics without microbiological confirmation in case of infection, and buying antibiotics without a prescription, as possible cause of antimicrobial resistance. Some HCW (28%; 13/47) reported having prescribed antibiotics because the patients asked for them; and a total of 15/47 (32%) responded that antibiotics could help patients recover faster when added to malaria treatment. Compared to nurses, most of the physicians recognised that excessive use of antibiotics without microbiological confirmation of infection could contribute to AMR spread (18/19 (95%) vs 19/28 (68%); *p* = 0.028).

**Conclusion:**

Most HCW recognised AMR as public health issue. However, a quarter of the participants did not know about the causes fostering the emergence of antimicrobial resistance. There is a need to perform regular HCW training in antimicrobial prescription, and to set up an antimicrobial stewardship program.

**Supplementary Information:**

The online version contains supplementary material available at 10.1186/s13756-022-01079-x.

## Background

Antimicrobial resistance (AMR) is challenging in low-income countries (LMICs) because of the high prevalence of infections and irrational use of antimicrobials [1–3]. Poor antibiotic stewardship due to lack of access to proper microbiology services and other essential diagnostic tools increases the AMR burden [4, 5].

The reduction of antimicrobial resistance requires rational use of antimicrobials, changes in prescription habits of healthcare workers (HCW), regulation of over-the-counter availability of antibiotics, improvements on hand hygiene, infection prevention and control [6]. Previous studies from Lambaréné and Libreville referral hospitals suggest a worrisome spread of antibiotic resistance in Gabon [7, 8]. In samples from a referral hospital in Libreville, half of the isolated *Klebsiella* spp. were gentamicin-resistant; 30%, were resistant to tazobactam, and 18–79% were resistant to commonly-used cephalosporin antibiotics (ceftazidime, cefotaxime, cefoxitin, cefuroxime, and cefalotin).There was a high fluoroquinolone resistance rate, ranging from 18% ciprofloxacin to 54% nalidixic acid resistance [8]. From Lambaréné, high resistance rates against routinely used antibiotics of gram-negative bacteria isolated in the Albert Schweitzer Hospital were reported already a decade ago [7].

Antimicrobial therapy is an essential part of infectious disease management, with a focus on time-dependent recognition, as any delay to first-dose antibiotic administration can be associated with increasing mortality [9]. Therefore, HCW need to govern appropriate knowledge, attitudes and practices towards antimicrobial prescription. Information on physicians’ and nurses’ knowledge and awareness on AMR will permit the development of effective interventions and containment of AMR. Surveys have been conducted to assess physicians’ knowledge and beliefs about antimicrobial use and resistance in developed countries [10, 11]; however, data on sub-Saharan African’s HCW awareness on AMR and antibiotic prescribing attitude such as Gabon is scarce. To date, there are no co-ordinated antimicrobial stewardship activities in Gabon. The routine and clinical data on microbiological resistance from the Lambaréné referral hospital show increasing AMR rates [7, 12]. We hypothesised that there is a gap in knowledge of HCW about AMR. To our knowledge, this is the first study undertaken to assess the knowledge and awareness about antimicrobial resistance among physicians and nurses in Lambaréné, Gabon. The findings from this study would be useful in implementing preventive and control interventions on AMR at regional and national levels.

## Methods

### Study design and setting

We conducted a cross-sectional survey among HCW at a Lambaréné referral hospital (Albert Schweitzer Hospital; HAS), the Centre de Recherches Médicales de Lambaréné (CERMEL), and peripheral first-level health facilities (n = 6) in Moyen-Ogooué province of Gabon; from February to June 2020. HAS is one of the regional referral hospitals, and the peripheral health facilities are primary care level facilities. CERMEL conducts infectious diseases research and manages infectious disease outpatients in collaboration with HAS [13]. Although the regional hospital has a microbiology laboratory, antimicrobials are often prescribed presumptively. Health facilities included in our study fully represent the primary and intermediate levels healthcare services available in Lambaréné.

### Survey instrument

We developed the questionnaire adapting content from previously published articles on the knowledge in HCW on AMR [11, 14]. The questionnaire collected information on HCW’s knowledge about antimicrobial resistance, attitudes about antibiotic prescribing, their perception of the importance of the problem of antimicrobial resistance, and their opinion about potential interventions designed to improve antibiotic use. The questionnaire was submitted in a pilot test to ten HCW to check comprehension and clarity of the questions (Additional file 1). The reliability of the questionnaire was tested, and the final questionnaire’s Cronbach’s alpha coefficient was 0.78.

### Data collection

The researchers went to the study site to inform, explain, and discuss the study with potential participants. Participants were approached individually, and invited to undertake the survey after signing the written consent form. All HCW who were antimicrobial prescribers were invited to participate in the study. The questionnaire was self-administered. To maintain the anonymity and to make sure that researchers were unable to accidentally identify the participants, we delegated one participant from each department (not having been part of the research team) to collect all questionnaires, and to convey them to the research team. The phone number of the research team was provided to the participants in case of additional clarification having been required regarding answers provided.

### Statistics

Study data were collected and entered by two independent data entry clerks into the Research Electronic Data Capture (RedCap) system [15], and any discrepancies were checked and corrected by a third individual. Percentages were calculated for categorical data. We used Fisher’s exact test to compare finding between nurses and physicians. A two-sided *p* value of < 0.05 was considered statistically significant. The analysis was performed using R studio version 4.0.2 (Integrated Development Environment for R, Boston) [16].

### Sample size

Due to the limited human resources of the health facilities of the Lambaréné region, all 53 individuals found to be eligible were invited to participate. We included all consenting HCW amongst those who were eligible (prescribers of antibiotics) and available.

## Results

### Participant characteristics

Of the 53 eligible health workers, 47 subjects accepted the invitation to participate and all of them filled the questionnaire. Twenty-four (53%) were female, 33 (70.2%) had obtained university education, while 14 (29.8%) had obtained secondary school level. The proportion of physicians was 40.42% (19/47). The median length of service of participants, i.e., how long the participant started working as a health worker, was 10.0 [IQR 4.3–16.8] years.

### Antimicrobial resistance spreading awareness and knowledge

More than half (64%; 30/47) of the prescribers responded that antimicrobial resistance is a problem in Gabon, while 30% (14/47) responded that it is a problem in their health facilities. A total of 70% (33/47) of health workers responded that there is uncoordinated antimicrobial prescription in their health facility; and 81% (38/47) responded that self-medication of antimicrobials could contribute to antimicrobial resistance. Educational level (*p* = 0.89), the type of health facility (*p* = 0.45), the professional category (*p* = 0.38), and the length of service (*p* = 0.15) were not associated with a better awareness of AMR. The overall knowledge about possible causes of antimicrobial resistance spread was limited. The attitudes most frequently selected by HCW to be associated with AMR spreading were ‘excessive use of antibiotics without microbiological confirmation of infection’ (37/47, 79%) and ‘buying antibiotics without a prescription’ (37/47, 79%). In general, the proportion of appropriate responses to the questions related to possible cause of AMR spread was higher among physicians than among nurses (Table 1). However, the difference was not statistically significant, except for the questions which asked whether the ‘excessive use of antibiotics without microbiological confirmation of infection could contribute to the spreading of AMR’ (94.7% vs. 67.9%, *p* = 0.028; Table 1).

**Table 1:** Comparison of knowledge and perception on antimicrobial resistance and infection control

Knowledge and perceptions on cause of antimicrobial resistance spreading^a^	Physicians (n = 19) [n (%)]	Nurses (n = 28) [n (%)]	*P* value
1. Too many broad-spectrum antibiotics prescriptions			0.5
No/I Do not Know	3 (16)	7 (25)	
Yes	16 (84)	21 (75)	
2. Long duration antibiotic treatments prescription			1.0
No/I Do not Know	7 (37)	10 (35)	
Yes	12 (63)	18 (65)	
3. Antibiotic therapy prescribed at doses that are too low			0.08
No/I Do not Know	2 (11)	10 (36)	
Yes	17 (89)	18 (64)	
4. Excessive use of antibiotics in case of suspicion of infection without confirmation			0.03
No/I Do not Know	1 (5)	9 (32)	
Yes	18 (95)	19 (68)	
5. Poor hand hygiene			0.5
No/I Do not Know	15 (79)	18 (64)	
Yes	4 (21)	10 (36)	
6. Buying antibiotics without a prescription			0.5
No/I Do not Know	3 (16)	7 (25)	
Yes	16 (84)	21 (75)	
7. Do not remove foreign material catheter prosthesis etc. that is the site of an infection			0.3
No/I Do not Know	12 (63)	12 (43)	
Yes	7 (37)	16 (57)	
8. Give too much credit to the speeches of medical representatives and pharmaceutical companies			0.6
No/I Do not Know	8 (42)	15 (54)	
Yes	11 (58)	13 (46)	

^a^Corrected answer expected for all question was “yes”

### Antimicrobial prescribing attitude and practice

**Fig. 1:**
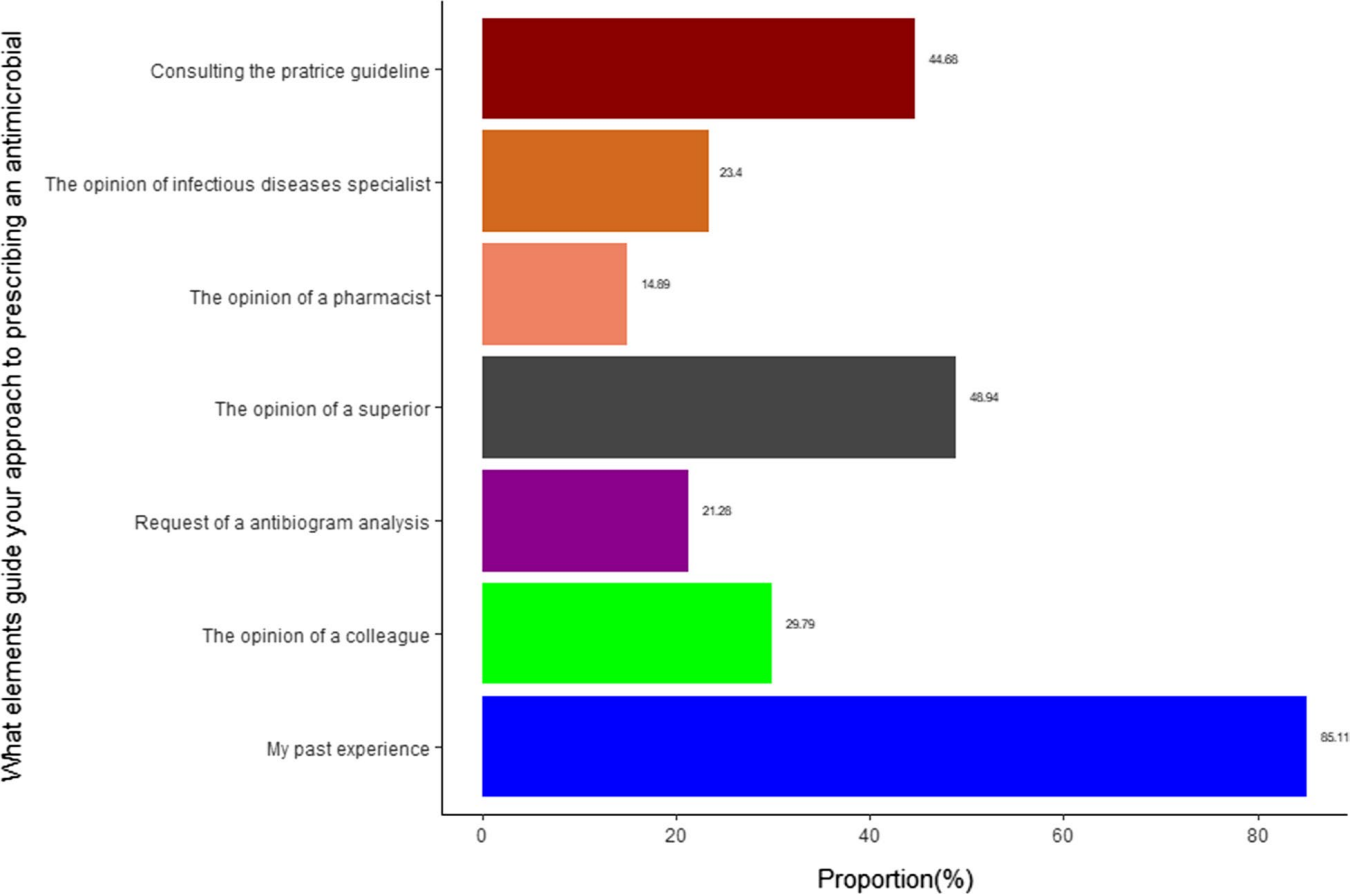
Elements influencing or guiding the approach to prescribing an antibiotic by the health workers interviewed

A total of 16/47 (35%) of participants across all professional groups prescribed at least three different groups of antibiotics during the week prior to the survey; 28% (13/47) reported to have prescribed antibiotics because the patients asked for them. The majority (55%; 26/47) of prescribers did not receive a recent training in antimicrobial prescription during the last year. Most of the HCW (85%, 40/47) referred to their own experience to choose the antibiotic to be prescribed to the patient; 21% (10/47) requested microbiological culture and antimicrobial susceptibility testing before prescribing antibiotics (Fig. 1). A total of 13/28 (46%) nurses versus 2/19 (10.5%) physicians (*p* = 0.023), reported that antibiotics, when added to malaria treatment, help the patients recover more quicker; 19/19 (100%) of physicians vs. 20/28(71%) nurses (*p* = 0.02) reported that antibiotics are always required for treating diarrhoea (Table 2).

**Table 2:** Comparison of Knowledge and Attitude about AMR and antibiotic use among healthcare workers

Attitudes toward antimicrobial prescribing^a^	Physicians n = 19 (%)	Nurses n = 28 (%)	*p* value
1. Antibiotics should be given to all patients with fever			1.0
No	18 (95)	26 (93)	
Yes	1 (5)	2 (7)	
2. Antibiotics are good for all patients with diarrhoea			0.02
No	0	20 (71)	
Yes	19 (100)	8 (28)	
3. Very expensive antibiotics must be stopped as soon as the patient is better			0.7
No	17 (90)	23 (82)	
Yes	2 (11)	5 (18)	
4. Any patient suspected of having tuberculosis should routinely receive ciprofloxacin while awaiting microscopy results			0.3
No	17 (90)	21 (75)	
Yes	2 (11)	7 (25)	
5. To strengthen tuberculosis treatment ciprofloxacin should be added to standard tuberculosis treatment			0.2
No	17 (90)	20 (71)	
Yes	2 (11)	8 (29)	
6. When deciding which antibiotic to use my choice depends more on expiration date availability than on the cause of infection			1.0
No	6 (32)	10 (36)	
Yes	13 (68)	18 (64)	
7. I believe it is difficult to choose the right antibiotic			0.6
No	11 (59)	13 (46)	
Yes	8 (42)	15 (54)	
8. In general, the prescription of short-term antimicrobials even without indication does not cause any harm in patients			1.0
No	16 (84)	23 (82)	
Yes	3 (16)	5 (18)	
9. Antibiotics help patients recover faster when added to malaria treatment			0.02
No	17 (90)	15 (54)	
Yes	2 (11)	13 (46)	

^a^The corrected answer expected for all questions was “No”

### Ratings of the helpfulness of potential interventions to improve antibiotic prescription

All of (47/47,100%) respondents strongly agreed that it would be very useful to perform regular training on antimicrobial prescriptions; and to have guidelines for antibiotic prescription; as well as that the vast majority agreed to determine the local antimicrobial resistance profile to guide empirical prescription 90% (42/47) (Table 3). Across all professional levels, HCW (91%; 43/47) agreed that setting up action to control the prescription of antimicrobials would be very useful in their health facility, but not to restrict the prescription of all antibiotics.

**Table 3:** Physicians’ and nurses’ beliefs on potential intervention to control AMR (N = 47)

Intervention to control antimicrobial resistance spreading	Very useful or useful n/47 (%)	Neutral n/47 (%)	Not useful/strongly not useful n/47 (%)
Organise training on the prescription of antibiotics	47 (100)	0 (0)	0
Provide local/national data on the proportion of bacteria resistant to the most commonly used antibiotics	42 (90)	1 (2)	4 (8)
Provide recommendations/practice guides/local/national protocols	47 (100)	0 (0)	0
Availability of the opinion of a bacteriologist	44 (94)	3 (6)	0
Availability of advice from colleagues with more experience in the field	40 (85)	5 (11)	2 (4)
Availability of the opinion of an infectious disease specialist	40 (85)	3 (6)	4 (9)
Availability of the opinion of a pharmacist	27 (57)	13 (28)	7 (15)
Availability of the operational hygiene team	27 (57)	9 (19)	11 (23)
Have access to computerized prescribing assistance	40 (85)	5 (11)	2 (4)
Restrict the prescription of certain antibiotics (then requiring specialist advice)	43 (91)	1 (2)	3 (6)
Restrict the prescription of all antibiotics	22 (47)	7 (15)	18 (38)
Regularly assess the prescription of antibiotics in a department, return the information to prescribers and possibly implement actions to improve the prescription	44 (94)	0 (0)	3 (6)

## Discussion

As per our knowledge, this is the first study assessing AMR knowledge and antibiotics prescription practice among HCW in Gabon.

Similar to other studies, the prescribers responded that antimicrobial resistance is a global and national public health challenge [17–19]. However, few participants recognised AMR as a problem in their health facilities. These findings are consistent with studies conducted in Ethiopia [20], Congo [21] and Sudan [22]. In contrast, studies from Peru [23] and Brazil [24] reported that the majority of prescribers perceived AMR as a problem in their health facilities. Study participants knew little about practices that contribute to the spreading of antimicrobial resistance. That could be explained by a lack of regular training on antimicrobial drugs prescription and AMR development. The lack of training contributes to antibiotic irrational prescription, and it is associated with AMR spread [1, 17, 25]. Fifteen HCW (32%) responded that antibiotics help patients recover faster when added to malaria treatment; a statement which should not be easily dismissed as incorrect, as a fair proportion of children (around 6.5%) with severe falciparum malaria in sub-Saharan Africa were reported to be concomitantly bacteraemic [20]. Rational antibiotics prescription is the main strategy to prevent AMR [6]. This is achieved by changing the prescribers’ attitude and practice. The results of this study suggest that misperceptions about antibiotic use prevailed, which may cause unnecessary prescription, as reported previously [26, 27]. According to this study, as in the study presented here, the antibiotics prescribers’ decision-making was sometimes influenced by patient requests (28%). Rodrigues et al. [28] and Asante et al. [14] reported similar findings. Patients’ influence on prescription of specific antibiotics is also a factor for excessive antibiotics prescription. Prescribers should explain the rationale of antibiotic prescriptions to the patients. Patients are sometimes not aware of the consequences of poor antibiotics prescription practices and AMR development [29, 30], with some of them pushing hard for prescription of antibiotics. Effective physician–patient communication and patient empowerment reduce antibiotics prescriptions [31]. Moreover, our findings stress the need to restrict the over-the-counter sale of certain antibiotics without a physician’s prescription.

Regarding potential interventions to combat AMR based on our findings, most favourite measures include: (1) to establish a national AMR surveillance program; and (2) to avail clinical microbiology laboratory capacity and local guidelines for rational use of antibiotics (Table 3); (3) to provide education on antimicrobial stewardship for health professional interventions. Using similar questionnaires, studies conducted in health workers from Saudi Arabia [25], and Ghana [14], and others focusing on junior doctors from Ethiopia [32], France and Scotland [11] suggested similar interventions. Only 30% (14/47) of the interviewed health workers responded that poor hand hygiene can contribute to AMR spread. This finding stresses the need of regular infection prevention and control training of health workers. A large percentage of healthcare-associated infections are preventable by improving hand hygiene practices [33]. Hand hygiene is an efficient infection prevention and control strategy in the fight against AMR transmission [34]. In fact, it is the key component of effective infection prevention and control programs recommended by WHO [35].

As expected, most physicians had a good attitude concerning the prescription of antimicrobials, compared to nurses. However, all of the physicians reported that antibiotics are always required for diarrhoea. This is an unexpected finding, which can be possibly explained by the fact that the most-frequently isolated pathogens in diarrhoea of children under five years in Lambaréné and surroundings were entero-invasive *Escherichia coli*/*Shigella* spp. (30.2%), enterotoxigenic *Escherichia coli* (24.6%), followed by *G. lamblia* (13.7%), *Cryptosporidium* spp. (12.9%), and rotavirus (9.5%) [36].

The strength of this study is that we included all level of health facilities in the Lambaréné region. Furthermore, this study provides valuable information on AMR and antimicrobial prescription, that could help to design appropriate local antimicrobial stewardship measures. Gabon has no formal AMR surveillance system as recommended by WHO [37]. It is important that a national or regional AMR surveillance be established to regularly update and disseminate AMR data to all HCW.

As with most surveys, our study does have certain limitations; for example, there is a possibility that respondents gave what was perceived as socially most acceptable answers. Furthermore, the sample size in our study is low compared with other studies conducted earlier in Africa reflecting the lack of HCW particularly outside the capitals in sub-Saharan Africa. We invited all HCW in the target health facilities to participate without restrictions. The small sample size of our study may limit the generalisability of our findings to national level, and the interpretation of our study results should be done with caution. There is a need to search for funding to conduct a national survey to further understand the issue of AMR in Gabon and to take appropriate actions to reduce its spread.

## Conclusions

Prescribers in Lambaréné recognise the AMR as a public health challenge in health facilities and in Gabon. Prescribers strongly believed that antimicrobial stewardship should be implemented in their health facilities to reduce the spread of AMR. There is a need to set up regular (re)training programs in antimicrobial prescription across health facilities in Lambaréné, Gabon.

## Supplementary Information

**Additional file 1.** Study questionnaire in English.

## Data Availability

All the questionnaires, and the datasets used and/or analysed during the current study are available from the corresponding author on reasonable request.

## Abbreviations

AMR: Antimicrobial resistance
CERMEL: ^1^Centre de Recherches Médicales de Lambaréné
HAS: Albert Schweitzer Hospital
HCW: Healthcare worker(s)
