# Supplementary material for: Knowledge and perception on antimicrobial resistance and antibiotics prescribing attitude among physicians and nurses in Lambaréné region, Gabon: a call for setting-up an antimicrobial stewardship program
Source: Antimicrob Resist Infect Control. 2022 Mar 3;11:44. doi: 10.1186/s13756-022-01079-x (PMC8892789; doi:10.1186/s13756-022-01079-x)
Supplement: Supplementary file 1 — Additional file 1. Study questionnaire in English. [file 13756_2022_1079_MOESM1_ESM.docx]

**Additional file 1.** Study questionnaire in English.

**Bacterial resistance and prescription of antibiotics**

***1-*** ***Professional information***

1. Sex : M [ ] F [ ]
2. Health facility -------------------------------
3. Hospital service: -----------------------------------------------------------
4. Type of health facility

☐Reference Hospital (HAS, CHRGRL)

☐Secondaire Hospital (CSUL)

☐First level hospital (dispensaire)

☐Research center

☐Other......................................

1. Education level: O primary O secondary O university
2. Profession: Doctor nurse assistant Other -----------------------
3. Length of medical career.............................................................................................

***Your practice***

**2. Have you prescribed an antibiotic in the past 6 months??** Yes  No

**3. How many antibiotics classes have you prescribed in the past 7 days?**

2  3 à 5  more than 5

**4.** **What elements influence or guide your approach to prescribing an antibiotic?**

|  | Check as many boxes as necessary |
| --- | --- |
| - Your past experience / your knowledge / the education you received |  |
| - The opinion of a colleague |  |
| - Request of a microbiology analysis and antibiogram - Opinion of a superior |  |
| - Opinion of a pharmacist |  |
| - Consulting international or national practice guides |  |

**5. Have you ever prescribed an antibiotic just to quell a patient's high demand, in order to satisfy their psychological need? Yes**  No

**6. Have you received training (s) on prescribing antibiotics during the past year?**

Yes  No

**If so, what type (s) of training was it?**

|  | Check as many boxes as necessary |
| --- | --- |
| - Law Lectures / conference |  |
| - Workshop (clinical cases…) |  |
| - Informal presentation in department |  |
| - Internet training |  |
| - Self-training - Visit of a medical representative - Medical staff of service |  |

***EVALUATION***

**A- *Knowledge - Resistance to antibiotics.***

**Please indicate whether the following situations seem to you to be more or less probable causes of resistance to antibiotic**

|  | Yes | No | I Do not Know |
| --- | --- | --- | --- |
| - Too many broad-spectrum antibiotics |  |  |  |
| - Long duration antibiotic treatments prescription |  |  |  |
| - Antibiotic therapy prescribed at doses that are too low |  |  |  |
| - Excessive use of antibiotics in any suspicion of infection without microbiological confirmation |  |  |  |
| - Poor hand hygiene |  |  |  |
| - Do not remove foreign material (catheter, prosthesis, etc.) that is the site of an infection |  |  |  |
| - Purchase without a medical prescription for antibiotics |  |  |  |
| - Give too much credit to the speeches of medical representatives and pharmaceutical companies |  |  |  |

***Problem of antibiotic resistance***

Yes No I do not Know

1. **Do you think that resistance to antibiotics**

**is a problem in Gabon?**

1. **Do you think that resistance to antibiotics?**
2. **Do you think that the way of using antibiotics currently in your hospital and other hospitals in Gabon can increase resistance to antibiotics?**

1. **Do you think that Antibiotics are overused in the community (self-medication)?**

***B-* *Prescription of antibiotics***

| **Using of antibiotic** | **Yes** | **No** | **I do not know** |
| --- | --- | --- | --- |
| Antibiotics should be given to all patients with fever |  |  |  |
| Antibiotics are good for all patients with diarrhoea |  |  |  |
| Very expensive antibiotics must be stopped as soon as the patient is better |  |  |  |
| Antibiotics help patients recover faster when added to malaria treatment |  |  |  |
| Any patient suspected of having tuberculosis should routinely receive ciprofloxacin while awaiting microscopy results |  |  |  |
| To strengthen tuberculosis treatment, ciprofloxacin should be added to standard tuberculosis treatment |  |  |  |
| I believe it is difficult to choose the right antibiotic |  |  |  |
| When deciding which antibiotic to use, my choice depends more on expiration date / availability than on the cause of infection. |  |  |  |
| In general, the prescription of short-term antimicrobials, even without indication, does not cause any harm in patients |  |  |  |

***C-*** ***Interventions to improve the prescription of antibiotics***

**Please indicate whether the following measures appear to you to be more or less useful in improving the prescription of antimicrobial .**

|  | | Very useful | Useful | | Neither useful nor unnecessary | Useless | Completely unnecessary |
| --- | --- | --- | --- | --- | --- | --- | --- |
| - Organize training on the prescription of antibiotics | |  |  | |  |  |  |
| - Provide recommendations / practice guides / local / national protocols | |  |  | |  |  |  |
| - Provide local / national data on the proportion of bacteria resistant to the most commonly used antibiotics | |  |  | |  |  |  |
| - Have access to computerized prescription assistance | |  |  | |  |  |  |
| - Availability of the opinion of a bacteriologist | |  |  | |  |  |  |
| - Availability of the opinion of an infectious disease specialist | |  |  | |  |  |  |
| - Availability of the opinion of a pharmacist | |  |  | |  |  |  |
| - Availability of the operational hygiene team | |  |  | |  |  |  |
| - Availability of advice from colleagues with more experience in the field | |  |  | |  |  |  |
| - Restrict the prescription of certain antibiotics (then requiring specialist advice) | |  |  | |  |  |  |
| - Restrict the prescription of all antibiotics | |  |  | |  |  |  |
| - Regularly assess the prescription of antibiotics in a department, return the information to prescribers and possibly implement actions to improve things | |  |  | |  |  |  |
|  |  | | |  |  |  |  |
